# The Impact of ChatGPT Exposure on User Interactions With a Motivational Interviewing Chatbot: Quasi-Experimental Study

**DOI:** 10.2196/56973

**Published:** 2025-03-21

**Authors:** Jiading Zhu, Alec Dong, Cindy Wang, Scott Veldhuizen, Mohamed Abdelwahab, Andrew Brown, Peter Selby, Jonathan Rose

**Affiliations:** 1 The Edward S. Rogers Sr. Department of Electrical & Computer Engineering, University of Toronto, Toronto, ON, Canada; 2 Department of Mechanical & Industrial Engineering, University of Toronto, Toronto, ON, Canada; 3 INTREPID Lab, Centre for Addiction and Mental Health, Toronto, ON, Canada; 4 Institute for Mental Health Policy Research, Centre for Addiction and Mental Health, Toronto, ON, Canada; 5 Department of Family and Community Medicine, University of Toronto, Toronto, ON, Canada; 6 Dalla Lana School of Public Health, University of Toronto, Toronto, ON, Canada

**Keywords:** chatbot, digital health, motivational interviewing, natural language processing, ChatGPT, large language models, artificial intelligence, experimental, smoking cessation, conversational agent

## Abstract

**Background:**

The worldwide introduction of ChatGPT in November 2022 may have changed how its users perceive and interact with other chatbots. This possibility may confound the comparison of responses to pre-ChatGPT and post-ChatGPT iterations of pre-existing chatbots, in turn affecting the direction of their evolution. Before the release of ChatGPT, we created a therapeutic chatbot, MIBot, whose goal is to use motivational interviewing to guide smokers toward making the decision to quit smoking. We were concerned that measurements going forward would not be comparable to those in the past, impacting the evaluation of future changes to the chatbot.

**Objective:**

The aim of the study is to explore changes in how users interact with MIBot after the release of ChatGPT and examine the relationship between these changes and users’ familiarity with ChatGPT.

**Methods:**

We compared user interactions with MIBot prior to ChatGPT’s release and 6 months after the release. Participants (N=143) were recruited through a web-based platform in November of 2022, prior to the release of ChatGPT, to converse with MIBot, in an experiment we refer to as MIBot (version 5.2). In May 2023, a set of (n=129) different participants were recruited to interact with the same version of MIBot and asked additional questions about their familiarity with ChatGPT, in the experiment called MIBot (version 5.2A). We used the Mann-Whitney *U* test to compare metrics between cohorts and Spearman rank correlation to assess relationships between familiarity with ChatGPT and other metrics within the MIBot (version 5.2A) cohort.

**Results:**

In total, 83(64.3%) participants in the MIBot (version 5.2A) cohort had used ChatGPT, with 66 (51.2%) using it on a regular basis. Satisfaction with MIBot was significantly lower in the post-ChatGPT cohort (*U*=11,331.0; *P*=.001), driven by a decrease in perceived empathy as measured by the Average Consultation and Relational Empathy Measure (*U*=10,838.0; *P*=.01). Familiarity with ChatGPT was positively correlated with average response length (ρ=0.181; *P*=.04) and change in perceived importance of quitting smoking (ρ=0.296; *P*<.001).

**Conclusions:**

The widespread reach of ChatGPT has changed how users interact with MIBot. Post-ChatGPT users are less satisfied with MIBot overall, particularly in terms of perceived empathy. However, users with greater familiarity with ChatGPT provide longer responses and demonstrated a greater increase in their perceived importance of quitting smoking after a session with MIBot. These findings suggest the need for chatbot developers to adapt to evolving user expectations in the era of advanced generative artificial intelligence.

## Introduction

### Background

Generative chatbots are conversational systems that provide synthesized replies using deep learning techniques [1]. In recent years, generative chatbots based on large language models (LLMs) have made major advancements in their ability to engage in natural and human-like conversations [2]. ChatGPT, a popular LLM-based generative chatbot developed by OpenAI [3], has demonstrated significant potential to be applied in public health and medicine for a variety of purposes such as providing health information, supporting patient education, summarizing clinical notes, and assisting in administrative tasks [4,5]. However, these applications have not typically focused on therapeutic interactions, such as those using motivational interviewing (MI) [6] techniques.

Prior to ChatGPT’s worldwide release in November 2022, our team developed MIBot [7], a therapeutic chatbot using MI techniques to guide smokers toward the decision to quit smoking. MIBot has a structured conversation flow, using scripted questions and LLM-generated MI-style reflections [6]. However, the popularity of ChatGPT and its advanced conversational capabilities raise questions about its influence on user interactions and expectations with simpler chatbots such as MIBot. These potential influences may in turn affect our future experiments with newer versions of MIBot, as the measurements used in future versions of MIBot may not be comparable to those in past versions, due to ChatGPT’s potential influences on participants.

### Related Work

Several recent studies have delved into the realm of generative chatbots, used in a medical or therapeutic context, and explored their effect on users. Perski et al [8] quantified the effect of the addition of a supportive chatbot to their smoking cessation application and found that it has increased user engagement and resulted in higher rates of quit success. Boucher et al [9] provided a comprehensive review of artificially intelligent chatbots in digital mental health interventions, discussing their acceptability, effects on user engagement and mental health outcomes, as well as their weaknesses and risks at the time, such as language proficiency and understanding. Moilanen et al [10] examined the effect of personality traits of a mental health chatbot on user engagement, finding that chatbots with a conscientious personality elicit the most user engagement and that users prefer their chatbot to be informative and confident rather than monotonic. Chow et al [11] framed ChatGPT as a disruptive technology and explored its impact on medical chatbots, claiming that it has the potential to improve access to health care services while expressing concerns over factors of risks such as reliability, transparency, privacy, and bias.

Together, these findings illustrate the growing influence of generative chatbots on user engagement and expectations in health-related contexts, particularly as advanced chatbots like ChatGPT redefine conversational norms. However, the impact of ChatGPT’s widespread use on users’ perceptions and behaviors toward other chatbots remains underexplored, raising questions about how exposure to such an advanced technology shifts user expectations and affects user behaviors. These findings served as important inspirations and motivations for this study, where we specifically investigate how users of one chatbot (MIBot) would change after being exposed to another chatbot (ChatGPT).

### Study Objective

This study aims to investigate how user interactions with MIBot have changed following the release of ChatGPT and examines the relationship between familiarity with ChatGPT and these changes. By comparing dialogue sessions with MIBot conducted before and after ChatGPT’s release, this study analyzes metrics such as average response length, satisfaction ratings, and effectiveness of the therapy. Additionally, we assess the relationship between participants’ familiarity with ChatGPT and these metrics to better understand the impact of ChatGPT on user behaviors and expectations. Findings from this study are expected to provide insights into how user expectations have evolved since the release of ChatGPT and inform future development strategies for chatbots in health-related applications.

## Methods

### Experiments

#### Overview

An experiment was conducted on November 18, 2022, about 2 weeks before the worldwide introduction of ChatGPT by OpenAI [3]. We then repeated that experiment on May 16, 2023, about half a year after ChatGPT was released. The 2 experiments used the exact same version of MIBot [7] but on different groups of independently recruited participants. From this part on, we will refer to the first experiment as MIBot (version 5.2) and the second experiment as MIBot (version 5.2A).

#### Overall Experiment Flow

The overall experiment flow strictly followed what was detailed in the original MIBot paper [7]. Here is a brief overview:

1. Recruitment: Participants were recruited through the Prolific [12] paid web-based recruitment system after providing consent.
2. Preconversation surveys: Participants filled out surveys about their smoking habits on a custom website, including metrics used in the filtering process.
3. Conversation initiation: MIBot initiated with a text chat about smoking, continuing only with participant consent.
4. Core conversation: The main chatbot conversation about smoking cessation, which contained prescripted questions, responses from the user, and then generated reflections from the chatbot, described in more detail in the Design of MIBot section.
5. Postconversation: Participants completed another readiness-to-quit survey, the Consultation and Relational Empathy (CARE) Measure [13], and additional qualitative questions.
6. Reporting: Completion of tasks was recorded in the Prolific system [12].
7. One-week-later survey: A week later, participants answered a follow-up survey, also including readiness-to-quit, the completion of which, along with passing a manual data quality review, was required for the participants to receive their payments.

#### Design of MIBot

MIBot asked 2 types of questions. We call the first type “main questions” (Textbox 1) and the second type “yes or no questions” (Textbox 2). The main questions were open-ended questions that promoted self-reflection within users about their smoking habits, while yes or no questions were close-ended questions posed after each generated reflection to find out whether or not the generated reflection made sense, was on topic, and was used as a transition to the next main question.

Textbox 1. **Main questions in MIBot conversations.**

1. To start, what is the thing you like most about smoking?
2. What else do you like about smoking?
3. Now, what is the thing you like least about smoking?
4. What else do you dislike about smoking?
5. Now, what is one thing about your smoking that you would like to change?
6. What will it look like when you have made this change in your smoking addiction?
7. Finally, what are the steps you need to take to make this change?

Textbox 2. **Yes or no questions in MIBot conversations.**

1. Did that make sense?
2. Did what I said make sense to you?
3. Does this make sense to you?

The overall conversation structure was fixed as follows:

1. Introductory statement about MIBot and asking for permission to talk about the participant’s smoking habits
2. Five to seven repetitions of
  - Main question
  - User response
  - Generated reflection
  - Yes or no question
  - User response
3. Conclusion and thanking the participant for their time

#### Smoking Status

The context of our study requires us to keep track of the smoking status of the participants, measured by the following three metrics:

1. User status: A label that denotes if a participant would have passed the screening from their preconversation survey imported from our previous MIBot studies [7]. If they have passed the screening, they are denoted as low confidence or discordant; if not, they are instead denoted as high confidence and not discordant.
2. Heaviness of Smoking Index [14]: A validated survey metric calculated from cigarettes per day and time to the first cigarette of the day. Heaviness of Smoking Index is an integer, where a higher value indicates a heavier smoking habit.
3. Quit attempts made: A binary value that denotes if a participant has made at least 1 quit attempt in the week leading up to the MIBot conversation.

#### MIBot Surveys

There were 3 surveys delivered to the participants from the original MIBot experiment flow: preconversation survey, postconversation survey, and 1-week-later survey. These surveys were used to determine how effective MIBot was in motivating smokers to make the decision to quit smoking as well as how participants felt about the conversation with MIBot.

The relevant parts of the survey for this study are the following:

1. The CARE Measure [13] consists of questions regarding the participant’s satisfaction with the conversation. Each question asks the participant to rate how well they think MIBot did on a scale from 1 to 5 or 0 if it does not apply. For example, participants were asked to evaluate MIBot’s ability to “make you feel at ease” or “letting you tell your ‘story’” back when they were interacting with MIBot. The CARE Measure is only included in the postconversation survey.
2. The Readiness Ruler [15] measures how ready the participant is to quit smoking. It consists of 3 ratings on a 0‐10 scale: how confident they are about quitting smoking now, how important it is for them to quit smoking, and how ready they feel to quit smoking now. The Readiness Ruler is included in all 3 surveys.
3. Two additional feedback questions, included only in the postconversation survey:
  - “What are 3 words that you would use to describe the chatbot?”
  - “What would you change about the conversation?”

#### ChatGPT Survey

To determine the extent of exposure to ChatGPT, for each participant in MIBot (version 5.2A), we included an additional short survey in the 1-week-later survey referred to as the ChatGPT survey. It contained 8 new questions designed to evaluate the participant’s knowledge and use of ChatGPT prior to engaging in MIBot (version 5.2A). The full ChatGPT survey can be found in Multimedia Appendix 1.

#### Recruitment and Data Inclusion

The steps taken for recruiting the participants for both MIBot (version 5.2) and MIBot (version 5.2A) were the exact same as detailed in the original MIBot paper [7]. Participants were recruited through the Prolific [12] web-based recruitment system, where they were informed by a recruitment description that they would engage in a text-based conversation with a chatbot designed to promote readiness to quit smoking, provide feedback on their experience, and complete the 1-week-later survey. The study was framed as an opportunity to contribute to research on chatbot-assisted smoking cessation, emphasized the confidentiality of participant data, and clearly stated that participation was voluntary, with the option to opt out if they did not agree to the terms. The entire study was delivered to the participants remotely, and they could participate in the study using their personal computers or mobile devices.

Notably, participants were screened using Prolific’s filters based on the following inclusion criteria: participants could be located in any country, were at least 18 years of age, were fluent in English, had a smoking status defined as either a current smoker (smoking at least 5 cigarettes a day for at least 1 year) or a recent smoker (smoking at least 5 cigarettes a day for less than 1 year), and had a minimum approval rate of 90% on their prior Prolific studies. Additionally, Prolific was set to recruit an equal number of male and female participants. However, due to additional screening conducted after recruitment, the final sample was not perfectly balanced by sex.

The data collected were manually reviewed for data inclusion following the same criteria as listed in the MIBot paper [7] except for 1 major difference. In the original MIBot study, we only included participants with either of the following qualities in their Readiness Ruler scores:

- Low confidence: confidence level less than or equal to 5
- Discordant: importance level more than 5 points below the confidence level

As the focus of this study is instead about ChatGPT’s influence on participants, we decided to include as many valid data entries as possible. Therefore, this filtering was not conducted in this study. As a result, the number of participants we included in this study increased from 100 to 143.

### Evaluation Metrics

To evaluate MIBot conversations and user behaviors from various aspects, we designed several evaluation metrics, for which detailed definitions are provided below.

#### Response Length

In this study, the key aspect of user behavior we focused on was the length of their responses to questions prompted by MIBot. Response length is an important indicator of user engagement and willingness to interact with the chatbot. Longer responses suggest that participants are more actively reflecting on the conversation, which aligns with the therapeutic goals of MI sessions.

We defined response length by its word count, excluding any punctuation. We also categorized the questions MIBot asks into 2 categories and looked at the user responses separately to gain more insight. As a result, there are three metrics we applied to measure the lengths of participant responses:

1. Average response length: average length of responses to all questions.
2. Average response length (main): average length of responses to main questions, which are the scripted questions MIBot asks to provoke contemplation of the users’ smoking habits.
3. Average response length (yes or no): average length of responses to yes or no questions, which are shown in Textbox 2.

#### Satisfaction With MIBot

Satisfaction with MIBot is a metric designed to measure user satisfaction after participating in a conversation with MIBot, a value between 0 and 1, calculated as the mean of the following three quantities:

1. Average CARE Measure (integers ranging from 1 to 5, in order of increasing satisfaction with MIBot), excluding answers where does not apply was selected. The mean is then rescaled to a number between 0 and 1 by subtracting 1 from the original value and dividing the result by 4.
2. Feedback sentiment score, based on answers to the first feedback question, “What are 3 words that you would use to describe the chatbot?” (integers ranging from 1 to 5, in order of increasing positivity). These scores were generated by automatically measuring the sentiment of the 3 words given by the user as feedback, described in the MIBot Survey section. The sentiment is computed using the neural network bert-base-multilingual-uncased-sentiment [16], which provides a score between 0 and 1.
3. MIBot improvement indicator, a binary value (0 or 1) if the participant indicated (in written feedback) that they would like MIBot improvement in response to the second feedback question, “What would you change about the conversation?” Responses are individually checked and manually labeled by a human collaborator. Since participants who suggested improvements are assumed to be less satisfied with MIBot, the value (1−MIBot improvement indicator) is used in the averaging calculation to get satisfaction with MIBot.

#### Effectiveness of MIBot

The goal of MIBot is to guide participants toward the decision to quit smoking by provoking contemplation. The more effective the conversation was, the more change we should observe in a participant. The effectiveness of MIBot is measured using 3 metrics related to the Readiness Ruler, as described in the MIBot Surveys section. The first of which, confidence change, is calculated as the difference between the confidence level before the conversation and the confidence level measured 1 week later, as recorded on the corresponding Readiness Rulers filled by the participants. Similarly, the importance change and readiness change were determined. As noted in the original MIBot paper [7], the primary outcome is the confidence change, as this is the most predictive of smoking cessation success [17-19].

#### Familiarity With ChatGPT

Familiarity with ChatGPT was given as a score between 0 and 126 (inclusive) to each participant based on their answers to the ChatGPT survey. Participants were first given a starting score of either 0 or 1: those who had not heard of ChatGPT were given a score of 0, and those who had were given a score of 1. Among those who had heard of ChatGPT, the score of those who had never used ChatGPT stayed at 1; for those who had used ChatGPT on top of knowing about it, they received an additional score according to the answers they had provided for 3 questions in the ChatGPT survey regarding their past use of ChatGPT. The three questions are as follows: (1) How long ago did you start using ChatGPT? (2) How frequently do you use ChatGPT? (3) On average, how long do your ChatGPT sessions last?

These 3 questions were presented as multiple-choice questions, the answers to which are presented in an ordered way and assigned a corresponding integer score from 1 to 5, as presented in Table 1. The final familiarity score they received was the product of the 3 integer scores, in addition to the 1 they already received. As a result, familiarity scores ranged from 0 to 126, with 0 being having never heard of ChatGPT, 1 being having heard of but never used ChatGPT, 2 being having used ChatGPT once a month or less for less than 5 minutes each session starting from less than a week ago, and 126 being having used ChatGPT multiple times per day with each session lasting longer than an hour for the past 5 months.

**Table 1.** Corresponding description for familiarity score assignment.

Corresponding score	Duration of use	Frequency of use	Length of each session
1	Less than a week	Once a month or less	Less than 5 minutes
2	Less than a month	Every 2 weeks	Less than 10 minutes
3	1‐3 months	Every week	10 to 30 minutes
4	3‐5 months	Every day	30 minutes to 1 hour
5	Over 5 months	Multiple times per day	More than 1 hour

### Statistical Analysis

#### Comparison of Pre- and Post-ChatGPT Cohorts

To capture the differences in behavior between participants in the 2 cohorts—November 2022 (MI version 5.2) and May 2023 (MI version 5.2A)—we used the Mann-Whitney *U* test to compare various metrics, such as average response length, satisfaction with MIBot, and changes in Readiness Ruler scores. This nonparametric test provided test statistics and *P* values for each metric, which are used to determine significant differences between the 2 groups. The test was done using the Python programming language (Python Software Foundation) with the pandas [20] and SciPy [21] libraries.

#### Relationships Between Familiarity With ChatGPT and Other Variables

To examine the impact of familiarity with ChatGPT within the MIBot (version 5.2A) cohort, we performed Spearman rank correlation analysis. This test assessed the relationships between participants’ familiarity scores (ranging from 0 to 126) and key metrics, such as average response length, satisfaction with MIBot, and changes in Readiness Ruler scores. Spearman rank correlation provided correlation coefficients and *P* values, indicating the strength and significance of each relationship. The test was also done using Python with the pandas [20] and SciPy [21] libraries.

### Ethical Considerations

This research was approved by the University of Toronto Research Ethics Board (protocol # 35567), as amended on June 29, 2022. Participants voluntarily provided informed consent by agreeing to all the terms stated in a consent form presented digitally to all participants during recruitment, fully outlining the study’s goals, procedures, potential risks, and privacy guarantees. It specified that no personally identifiable information would be collected, and any inadvertent identifiers would be removed prior to analysis or publication. Participants received a total of US $6.25 for MIBot (version 5.2) and US $6.18 for MIBot (version 5.2A) as compensation for completing all tasks in the study, and partial compensation was not provided to those who did not complete all components of the experiment. No identifiable participant information is included in any images or supplementary materials in the manuscript.

## Results

### Participant Demographic

Participant demographic data of both MIBot (version 5.2) and MIBot (version 5.2A) are listed in Tables2  3, including categorical and continuous variables, respectively. Additional data of the demographics of the participants in both MIBot (version 5.2) and MIBot (version 5.2A) are listed in Multimedia Appendix 2.

**Table 2.** Categorical demographic data of participants in cohort MIBot (version 5.2; November 2022, pre-ChatGPT) and MIBot (version 5.2A; May 2023, post-ChatGPT).

Characteristic		MIBot (version 5.2) (n=143), n (%)	MIBot (version 5.2A) (n=129), n (%)
**Sex**			
	Male	72 (50.3)	63 (48.8)
	Female	71 (49.7)	66 (51.2)
**User status**			
	Low confidence or discordant	100 (69.9)	91 (70.5)
	High confidence and not discordant	43 (30.1)	38 (29.5)
**Smoking status**			
	I am a current smoker (smoke at least 5 cigarettes a day and have smoked this amount for at least 1 year)	121 (84.6)	107 (82.9)
	I am a recent smoker (smoke at least 5 cigarettes a day and have smoked this amount for less than 1 year)	22 (15.4)	22 (17.1)
**Quit attempts made**			
	Yes	69 (48.3)	46 (35.7)
	No	74 (51.7)	83 (64.3)

**Table 3.** Continuous demographic data of participants in cohort MIBot (version 5.2; November 2022, pre-ChatGPT) and MIBot (version 5.2A; May 2023, post-ChatGPT).

Characteristic	MIBot (version 5.2)		MIBot (version 5.2A)	
Mean (SD)	Median (IQR)	Mean (SD)	Median (IQR)
Age (years)	29.22 (9.69)	26.00 (23.00‐33.00)	32.75 (11.36)	29.00 (24.00‐39.00)
HSI^a^	1.45 (1.38)	1.00 (0.00‐2.00)	1.67 (1.45)	1.00 (0.00‐3.00)
**Readiness Rulers, preconversation**				
Confidence	4.52 (2.75)	4.00 (2.00‐7.00)	4.12 (2.60)	4.00 (2.00‐6.00)
Importance	5.84 (2.84)	6.00 (3.00‐8.00)	5.60 (2.65)	6.00 (3.00‐8.00)
Readiness	5.36 (2.83)	5.00 (3.00‐8.00)	4.79 (2.58)	5.00 (3.00‐7.00)

a HSI: Heaviness of Smoking Index.

### Reach of ChatGPT

The familiarity score, described in the Familiarity With ChatGPT section, provides an estimate of the reach of ChatGPT to the public. Table 4 provides a more detailed description of the distribution of the familiarity score, where we bin the participants based on familiarity score ranges and assign labels to each score range. “Unexposed” is the group of participants who have not heard of ChatGPT prior to this study. “Aware” denotes that the participants have heard of ChatGPT but did not use it in any capacity. People who have used ChatGPT are separated into 3 groups, which are “casual user,” “consistent user,” and “dedicated user,” based on their familiarity score.

**Table 4.** Distribution of ChatGPT familiarity levels among participants in the MIBot (version 5.2A) cohort.

ChatGPT familiarity level	Count, n (%)	Score
Unexposed	12 (9.3)	0
Aware	34 (26.4)	1
Casual user	17 (13.2)	2 to 8
Consistent user	37 (28.7)	9 to 27
Dedicated user	29 (22.5)	28 or higher

### Comparison of Pre- and Post-ChatGPT Cohorts

Table 5 shows the Mann-Whitney *U* test results that compare between participants from the November 2022 (MI version 5.2) and May 2023 (MI version 5.2A) cohorts. In terms of average response length, there was no significant difference between the 2 cohorts, whether looking at all responses together or breaking them down into responses to main questions and responses to yes or no questions. Satisfaction with MIBot was significantly higher in MIBot (version 5.2) than in MIBot (version 5.2A; *U*=11,331.0; *P*=.001). Specifically, of the 3 metrics satisfaction with MIBot was aggregated of, Average CARE Measure was the only one showing any significant difference, with it being significantly higher in MIBot (version 5.2) compared to MIBot (version 5.2A; *U*=10,838; *P*=.01). As for changes in the Readiness Ruler, no significant differences were found between the cohorts in terms of confidence change, importance change, or readiness change.

**Table 5.** Results of the Mann-Whitney *U* test comparing key metrics between cohort MIBot (version 5.2; pre-ChatGPT) and MIBot (version 5.2A; post-ChatGPT).

Variable		MIBot (version 5.2)		MIBot (version 5.2A)		Test statistic (*U*)	*P* value
		Mean (SD)	Median (IQR)	Mean (SD)	Median (IQR)		
**Response length**							
	Average response length	4.98 (2.49)	4.46 (3.31‐5.89)	5.36 (3.54)	4.29 (3.13‐7.00)	9248.0	.97
	Average response length (main)	8.18 (4.00)	7.60 (5.50‐9.69)	8.04 (4.71)	6.62 (4.86‐10.43)	9936.5	.27
	Average response length (yes or no)	2.02 (1.91)	1.00 (1.00‐2.07)	2.44 (3.04)	1.17 (1.00‐2.80)	8301.5	.13
**Satisfaction scores**							
	Satisfaction with MIBot	0.74 (0.22)	0.85 (0.52‐0.91)	0.66 (0.21)	0.61 (0.49‐0.85)	11,331.0	.001
	Average CARE^a^ Measure	0.77 (0.16)	0.78 (0.66‐0.90)	0.72 (0.17)	0.74 (0.58‐0.84)	10,838.0	.01
	MIBot improvement indicator	0.57 (1.01)	0.00 (0.00‐1.00)	0.76 (1.12)	0.00 (0.00‐1.00)	8192.5	.06
	Feedback sentiment score	0.79 (0.15)	0.80 (0.80‐0.80)	0.76 (0.18)	0.80 (0.80‐0.80)	10,028.0	.15
**Readiness Ruler**							
	Confidence change	0.86 (2.32)	1.00 (0.00‐2.00)	0.60 (2.08)	0.00 (0.00‐2.00)	9893.0	.29
	Importance change	0.69 (1.85)	0.00 (0.00‐2.00)	0.40 (1.98)	0.00 (0.00‐1.00)	10,003.5	.21
	Readiness change	0.35 (1.82)	0.00 (−1.00 to 1.00)	0.33 (2.07)	0.00 (−1.00 to 1.00)	9260.0	.95

a CARE: Consultation and Relational Empathy.

### Relationships Between Familiarity With ChatGPT and Other Variables

Table 6 shows the Spearman rank correlation results evaluating relationships between familiarity with ChatGPT and various other variables within the MIBot (version 5.2A) cohort. In terms of average response length, there was a significant positive correlation between familiarity with ChatGPT and overall average response length (ρ=0.181; *P*=.04) as well as with average response length for main questions (ρ=0.180; *P*=.04) and average response length for yes or no questions (ρ=0.197; *P*=.03). Satisfaction with MIBot had a weak negative correlation with familiarity score, but it was not statistically significant (ρ=−0.171; *P*=.05). Of the 3 metrics aggregated into satisfaction with MIBot, only the MIBot improvement indicator showed a significant positive correlation with familiarity with ChatGPT (ρ=0.188; *P*=.03). Among the Readiness Ruler changes, a significant positive correlation was observed between familiarity with ChatGPT and importance change (ρ=0.296; *P*<.001) but not for confidence change or readiness change.

**Table 6.** Spearman rank correlation analysis for the MIBot (version 5.2A) cohort.

Variable		Familiarity with ChatGPT	
		ρ	*P* value
**Response length**			
	Average response length	0.181	.04
	Average response length (main)	0.180	.04
	Average response length (yes or no)	0.197	.03
**Satisfaction scores**			
	Satisfaction with MIBot	−0.171	.05
	Average CARE^a^ Measure	−0.112	.21
	MIBot improvement indicator	0.188	.03
	Feedback sentiment score	−0.023	.80
**Readiness Ruler**			
	Confidence change	0.165	.06
	Importance change	0.296	<.001
	Readiness change	0.078	.38

a CARE: Consultation and Relational Empathy.

## Discussion

### Principal Findings

This study aimed to investigate how user interactions with MIBot changed following the release of ChatGPT as well as the relationship between ChatGPT familiarity and these changes. From the results, we found that users interacting with MIBot after the release of ChatGPT were less satisfied with the chatbot, particularly in terms of perceived empathy, but users more familiar with ChatGPT provided longer responses and showed greater increase in their perceived importance of quitting smoking.

The premise of this study is that people have been exposed to ChatGPT due to its high popularity, which is supported by the distribution of familiarity scores in the MIBot (version 5.2A) cohort. From Table 4, ChatGPT is observed to have a widespread reach, with only 12 (9.3%) participants of MIBot (version 5.2A) having never heard of it, and 34 (26.4%) having only learned about it but never used it. Collectively, 83 (64.3%) participants have used ChatGPT at least to some capacity, most of whom are consistent or even dedicated users, indicating that the public has received massive exposure to the popular chatbot.

Using the Mann-Whitney *U* test (Table 5) to compare participants from the MIBot (version 5.2) and MIBot (version 5.2A) cohorts, we observed that most variables did not show significant differences between the groups. However, satisfaction with MIBot was significantly lower in the MIBot (version 5.2A) cohort (*U*=11,331.0; *P*=.001), a finding primarily driven by differences in the Average CARE Measure. This specific component of the satisfaction score, which reflects perceived empathy in interactions, was significantly higher in the MIBot (version 5.2) cohort (*U*=10,838.0; *P*=.01). These findings suggest that participants in the post-ChatGPT cohort, many of whom were familiar with or at least aware of ChatGPT’s advanced conversational capabilities, may have developed higher expectations for chatbot empathy, which MIBot may not fully meet.

Within the MIBot (version 5.2A) cohort, Spearman rank correlation analysis revealed that familiarity with ChatGPT was positively correlated with average response length across all response types, indicating that more experienced users were likely to engage in longer interactions. However, while users who are more familiar with ChatGPT are more likely to suggest improvements for MIBot (ρ=0.188; *P*=.03), satisfaction with MIBot as a whole had only a weak, nonsignificant negative correlation with familiarity (ρ=−0.171; *P*=.05), even though the *U* test results showed that satisfaction was significantly lower in the MIBot (version 5.2A) cohort. This discrepancy suggests that while ChatGPT exposure may influence satisfaction overall, familiarity alone does not predict satisfaction levels. It may be that other factors unique to the MIBot (version 5.2A) cohort, such as general exposure to more advanced conversational artificial intelligence or broader trends in technology expectations, are impacting satisfaction with MIBot in a way that the specific familiarity score we designed failed to capture. In other words, the drop in satisfaction with MIBot might reflect a general shift in expectations for chatbot performance rather than an effect specific to individual familiarity with ChatGPT.

Interestingly, although familiarity with ChatGPT appears to make users more critical of MIBot’s capabilities, it is also associated with an increase in participants’ perceived importance of quitting smoking (ρ=0.296; *P*<.001), a key measure of MIBot’s effectiveness. This seemingly paradoxical finding could suggest that while exposure to advanced generative chatbots raises expectations for technical quality, it might also enhance users’ receptiveness to the broader goals of MIBot, such as helping users resolve ambivalence and guiding them toward change, and as a result, making a less advanced chatbot more effective.

### Practical Implications

The findings from this study suggest several practical implications for future chatbot development, especially in health-related applications. The decrease in satisfaction with MIBot from post-ChatGPT users highlights a need for researchers and developers to adapt to higher expectations of chatbots, especially for empathy. Additionally, the tendency of users familiar with ChatGPT to provide longer, more detailed responses suggests that chatbots should be capable of handling more nuanced interactions. Finally, the positive correlation between ChatGPT familiarity and MIBot’s effectiveness in increasing the perceived importance of quitting smoking implies that users more experienced with advanced generative chatbots may have more trust in the technology and be more receptive to interactions guiding themselves toward change.

### Comparison With Prior Works

Unlike prior studies that examined and reviewed various aspects of chatbots that may affect user engagement and effectiveness in different contexts [8-10], this study specifically and uniquely investigates how exposure to and familiarity with a more advanced chatbot (ChatGPT) affect user interactions with a simpler chatbot (MIBot). Building on to our previous work, MIBot [7], our new experiments suggest that MIBot continues to increase users’ readiness to quit even after the introduction of ChatGPT. Nevertheless, overall user satisfaction has decreased, possibly affected by higher user expectations among those exposed to ChatGPT. This could be a disruptive effect from ChatGPT as predicted by Chow et al [11]. Moreover, our findings also provide unique insights not found in related prior works, such as that familiarity with ChatGPT positively correlates with core metrics for user engagement with and effectiveness of MIBot, a simpler chatbot than ChatGPT. It also presented a measurement, at a specific point in time, of the exposure level to ChatGPT among our recruited participants, which demonstrates the extent of ChatGPT’s reach at that time.

### Limitations

There are several limitations regarding this study. Since we followed the same experiment flow as the original MIBot paper [7] and used mostly the same evaluation metrics, the limitations of the MIBot experiments documented in the original MIBot paper [7] also apply to this study. Moreover, there exists an additional type of sampling bias specific to this study. Since for both of our experiments, we recruited participants through Prolific [12], a web-based recruitment system, it is possible that these participants are more fluent with technology than the general public and therefore more likely to have been exposed to ChatGPT or have the effects of ChatGPT manifest in a way that does not fairly represent a more general population. Furthermore, our 2 experiments used different groups of people with some demographic differences, notably with a difference in their average age, which may result in different smoking behaviors and success rates of smoking cessation [22,23]. Finally, it is important to acknowledge the possibility of confounding factors not accounted for in our study, which may have influenced how participants interacted with MIBot or perceived its effectiveness, along with familiarity with ChatGPT.

### Conclusions

Given MIBot, a generative chatbot for smoking cessation less advanced than ChatGPT, this study aimed to find any potential changes to how users interact with MIBot by comparing user interactions before and after ChatGPT’s release. We found that post-ChatGPT users are less satisfied with MIBot overall, particularly in terms of perceived empathy. Moreover, as users gain more familiarity with ChatGPT, they provide longer responses and show a greater increase in their perceived importance of quitting smoking. These findings suggest the need for continuous innovation in chatbot technology, particularly in digital health, to meet the evolving expectations of users accustomed to more advanced chatbot interactions.

## Supplementary material

10.2196/56973 Multimedia Appendix 1 Questions related to ChatGPT on the week-later survey.

10.2196/56973 Multimedia Appendix 2 Additional demographics data.

## Abbreviations

CARE: Consultation and Relational Empathy
LLM: large language model
MI: motivational interviewing
