# Supplementary material for: The Impact of ChatGPT Exposure on User Interactions With a Motivational Interviewing Chatbot: Quasi-Experimental Study
Source: JMIR Form Res. 2025 Mar 21;9:e56973. doi: 10.2196/56973 (PMC11952273; doi:10.2196/56973)
Supplement: Multimedia Appendix 1 [file formative-v9-e56973-s001.docx]

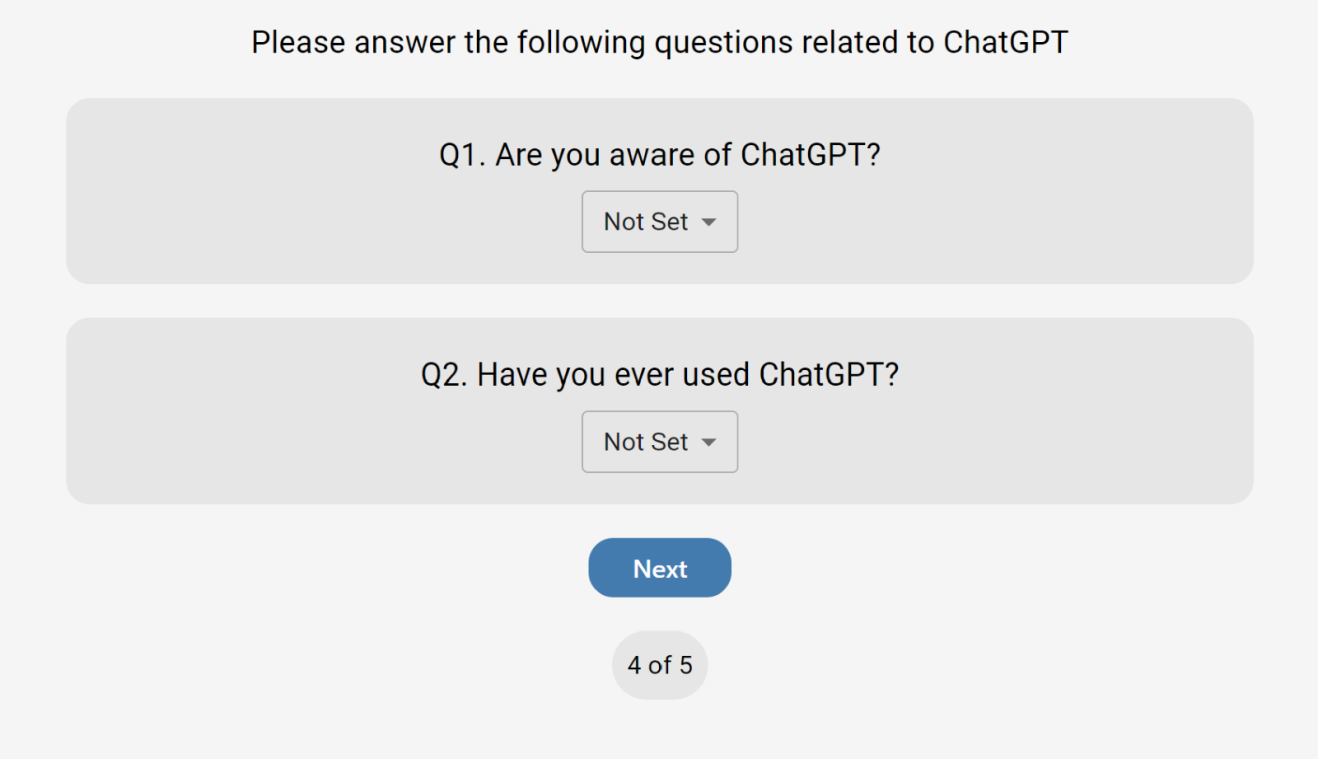


**Figure S1.** Screen that measures ChatGPT familiarity.


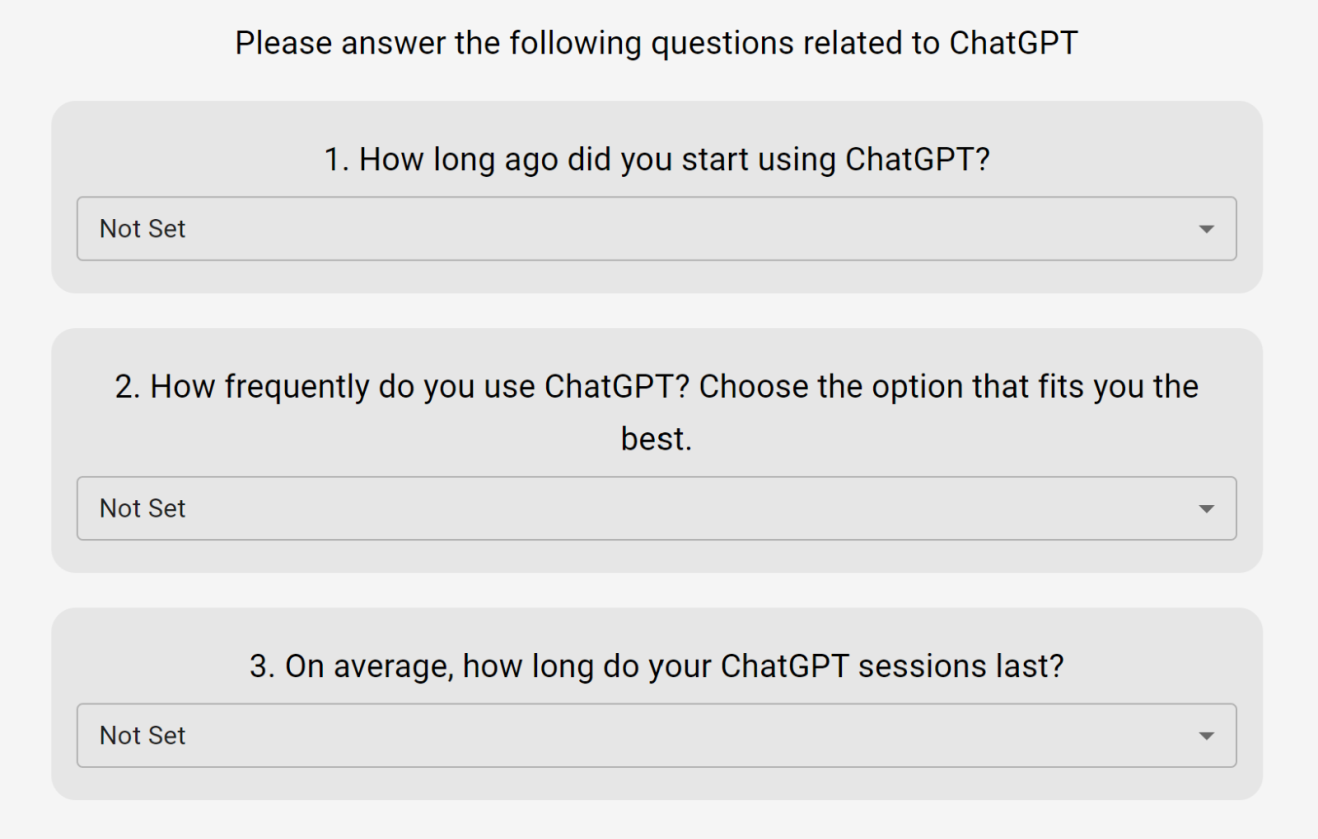


**Figure S2**. Screen that measures ChatGPT familiarity continued.


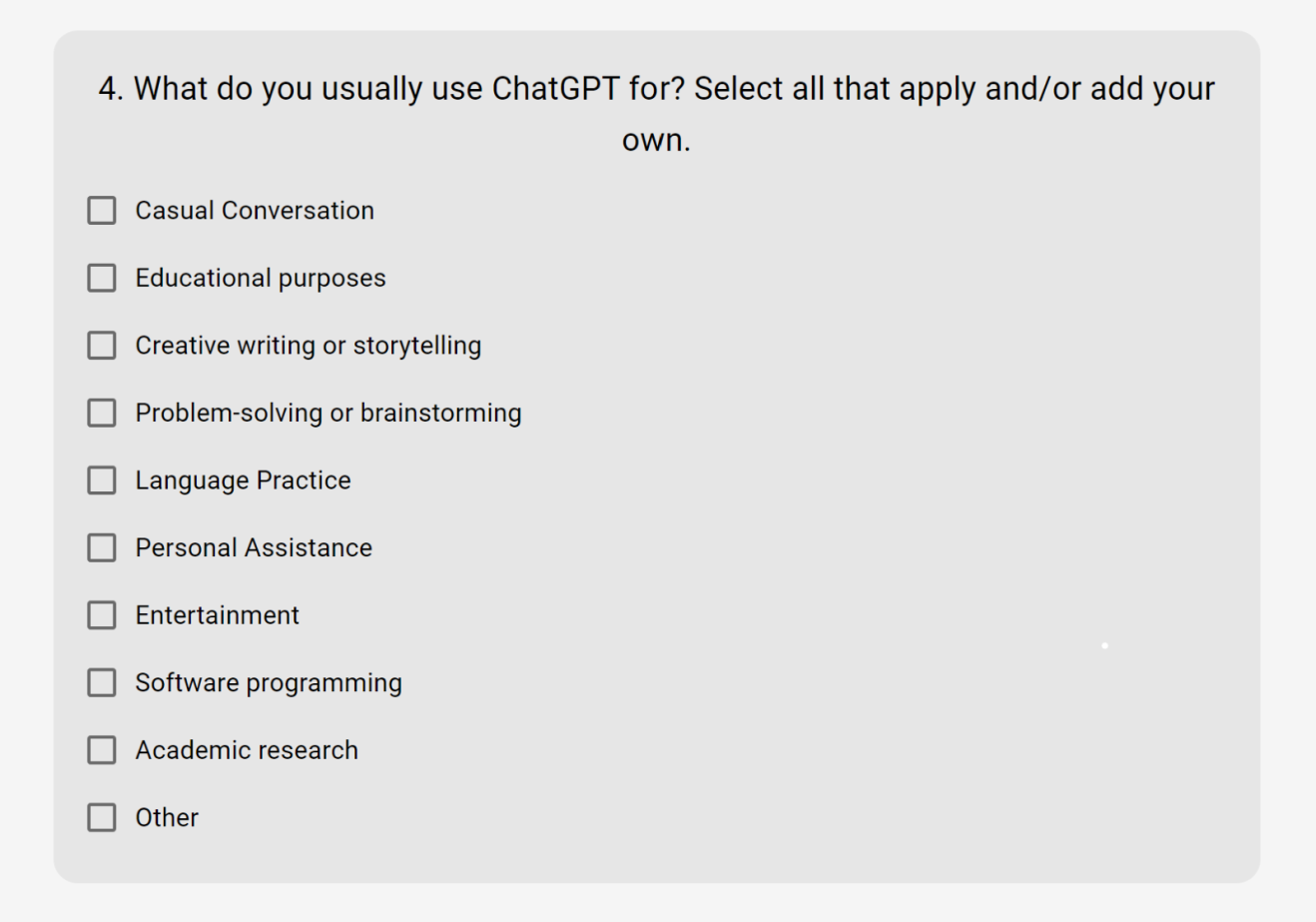


**Figure S3**. Additional questions for ChatGPT usage.


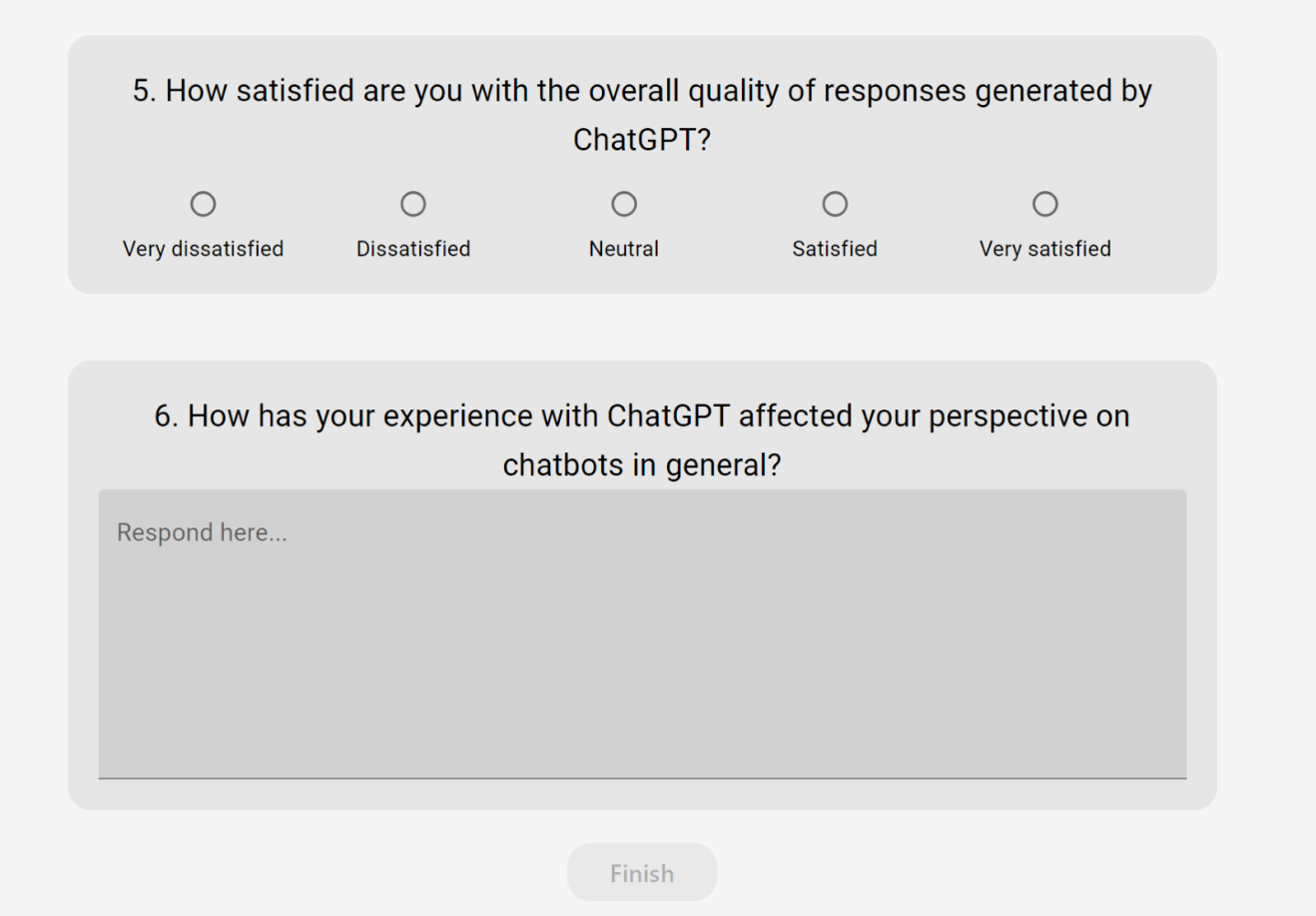


**Figure S4**. Additional questions for ChatGPT related feedback.
