# Supplementary material for: The Impact of ChatGPT Exposure on User Interactions With a Motivational Interviewing Chatbot: Quasi-Experimental Study
Source: JMIR Form Res. 2025 Mar 21;9:e56973. doi: 10.2196/56973 (PMC11952273; doi:10.2196/56973)
Supplement: Multimedia Appendix 2 [file formative-v9-e56973-s002.docx]

| **Characteristic, Count, n (%)** | | **MI v5.2 (n=143)** | **MI v5.2A (n=129)** |
| --- | --- | --- | --- |
| **Age Distribution** |  |  |  |
|  | 18 to 19 | 3 (2.1) | 0 (0) |
|  | 20 to 29 | 87 (60.8) | 68 (52.7) |
|  | 30 to 39 | 37 (25.9) | 29 (22.5) |
|  | 40 to 49 | 10 (7.0) | 17 (13.2) |
|  | 50 to 59 | 4 (2.8) | 13 (10.1) |
|  | ≥ 60 | 2 (1.4) | 2 (1.6) |
| **Country of residence** |  |  |  |
|  | Australia | 3 (2.1) | 1 (0.8) |
|  | Austria | 0 (0) | 1 (0.8) |
|  | Canada | 2 (1.4) | 5 (3.9) |
|  | Chile | 4 (2.8) | 1 (0.8) |
|  | Czech Republic | 0 (0) | 1 (0.8) |
|  | Denmark | 0 (0) | 1 (0.8) |
|  | Estonia | 1 (0.7) | 0 (0) |
|  | Finland | 1 (0.7) | 1 (0.8) |
|  | Germany | 0 (0) | 2 (1.6) |
|  | Greece | 8 (5.6) | 8 (6.2) |
|  | Hungary | 0 (0) | 2 (1.6) |
|  | Ireland | 1 (0.7) | 0 (0) |
|  | Israel | 0 (0) | 1 (0.8) |
|  | Italy | 4 (2.8) | 6 (4.7) |
|  | Japan | 0 (0) | 1 (0.8) |
|  | Latvia | 1 (0.7) | 0 (0) |
|  | Mexico | 17 (11.9) | 8 (6.2) |
|  | Netherlands | 2 (1.4) | 2 (1.6) |
|  | New Zealand | 0 (0) | 1 (0.8) |
|  | Norway | 1 (0.7) | 0 (0) |
|  | Poland | 17 (11.9) | 19 (14.7) |
|  | Portugal | 23 (16.1) | 18 (14.0) |
|  | Slovenia | 2 (1.4) | 2 (1.6) |
|  | South Africa | 34 (23.8) | 19 (14.7) |
|  | Spain | 6 (4.2) | 5 (3.9) |
|  | Sweden | 2 (1.4) | 2 (1.6) |
|  | United Kingdom | 10 (7.0) | 15 (11.6) |
|  | United States | 4 (2.8) | 7 (5.4) |
| **Ethnicity** |  |  |  |
|  | Asian | 0 (0) | 3 (2.3) |
|  | Black | 32 (22.4) | 12 (9.3) |
|  | White | 89 (62.2) | 97 (75.2) |
|  | Mixed | 14 (9.8) | 10 (7.8) |
|  | Other | 8 (5.6) | 7 (5.4) |
| **First Language** |  |  |  |
|  | Afrikaans | 1 (0.7) | 1 (0.8) |
|  | Dutch | 2 (1.4) | 1 (0.8) |
|  | English | 56 (39.2) | 49 (38.0) |
|  | Estonian | 1 (0.7) | 0 (0) |
|  | Finnish | 1 (0.7) | 1 (0.8) |
|  | French | 1 (0.7) | 0 (0) |
|  | German | 0 (0) | 3 (2.3) |
|  | Greek | 9 (6.3) | 8 (6.2) |
|  | Hungarian | 0 (0) | 3 (2.3) |
|  | Italian | 5 (3.5) | 4 (3.1) |
|  | Japanese | 0 (0) | 1 (0.8) |
|  | Latvian | 1 (0.7) | 0 (0) |
|  | Norwegian | 1 (0.7) | 0 (0) |
|  | Polish | 16 (11.2) | 20 (15.5) |
|  | Portuguese | 20 (14.0) | 18 (14.0) |
|  | Russian | 1 (0.7) | 1 (0.8) |
|  | Slovenian | 1 (0.7) | 2 (1.6) |
|  | Spanish | 24 (16.8) | 12 (9.3) |
|  | Swahili | 1 (0.7) | 0 (0) |
|  | Swedish | 1 (0.7) | 3 (2.3) |
|  | Turkish | 0 (0) | 2 (1.6) |
|  | Other | 1 (0.7) | 0 (0) |
| **Employment status** |  |  |  |
|  | Full-Time | 64 (44.8) | 59 (45.7) |
|  | Part-Time | 26 (18.2) | 20 (15.5) |
|  | Unemployed (and job seeking) | 27 (18.9) | 21 (16.3) |
|  | Not in paid work (e.g. homemaker', 'retired or disabled) | 5 (3.5) | 8 (6.2) |
|  | Due to start a new job within the next month | 3 (2.1) | 4 (3.1) |
|  | Other | 10 (7.0) | 9 (7.0) |
|  | N/A | 8 (5.6) | 8 (6.2) |
| **Student Status** |  |  |  |
|  | Yes | 71 (49.7) | 51 (39.5) |
|  | No | 63 (44.1) | 70 (54.3) |
|  | N/A | 9 (6.3) | 8 (6.2) |
